# An Experimental Education Project for Consultations of Older Adults during the Pandemic and Healthcare Lockdown

**DOI:** 10.3390/healthcare9040425

**Published:** 2021-04-06

**Authors:** Agnieszka Neumann-Podczaska, Mikołaj Seostianin, Konrad Madejczyk, Piotr Merks, Urszula Religioni, Zofia Tomczak, Sławomir Tobis, Daniela Claudia Moga, Melody Ryan, Katarzyna Wieczorowska-Tobis

**Affiliations:** 1 Chair and Department of Palliative Medicine, Poznan University of Medical Sciences, 61-245 Poznan, Poland; ar-n@wp.pl (A.N.-P.); mseostianin@gmail.com (M.S.); Konrad_madejczyk@wp.pl (K.M.); z.t.tomczak2@gmail.com (Z.T.); kwt@tobis.pl (K.W.-T.); 2 Collegium Medicum, Faculty of Medicine, Cardinal Stefan Wyszyński University, 01-938 Warsaw, Poland; piotrmerks@googlemail.com; 3 Collegium of Business Administration, Warsaw School of Economics, 02-513 Warsaw, Poland; 4 Department of Occupational Therapy, Poznan University of Medical Sciences, 60-781 Poznan, Poland; stobis@gmail.com; 5 Department of Pharmacy Practice and Science, College of Pharmacy, University of Kentucky, Lexington, KY 40536, USA; daniela.moga@uky.edu (D.C.M.); melody.ryan@uky.edu (M.R.); 6 Department of Epidemiology, College of Public Health, University of Kentucky, Lexington, KY 40536, USA; 7 Sanders-Brown Center on Aging, University of Kentucky, Lexington, KY 40504, USA

**Keywords:** interprofessional education, medical students, pharmacy students, telehealth, COVID-19, older adults

## Abstract

Objective: To develop a mentor-supervised, interprofessional, geriatric telemedicine experiential education project in response to the COVID-19 pandemic. Method: Medical and pharmacy students collaborated via remote consultations to address the coexistence of multimorbidity and polypharmacy in geriatric patients. In-depth interviews of students and patients as well as Likert scale-based telephonic survey were performed for a comprehensive evaluation of the project’s significance. Results: To date, 49 consultations have been conducted. Remote consultations performed by medical and pharmacy students working collaboratively were beneficial for both students, participants. Conclusions and Practice Implications: This experimental education project provided students with authentic challenges while simultaneously delivering care to the older adults who are susceptible to disruption of care associated with the pandemic. Further development and expanded implementation of such approaches may be a post-pandemic practice to provide more accessible care for senior patients while incorporating interprofessional education.

## 1. Background

Coronavirus Disease 2019 (COVID-19) rapidly spread throughout the world, causing a global pandemic [1]. The restrictions imposed upon its outbreak impacted many healthcare establishments, which were either closed to non-emergency cases or entirely devoted to treating COVID-19 patients [1]. Decreased healthcare accessibility during the pandemic transformed the management of many chronic conditions by limiting outpatient visits in order to reduce the exposure to novel coronavirus and relying on telemedicine to provide continued patient care [2,3].

Social distancing measures and the workforce’s involvement in controlling the crisis also imposed changes in the way healthcare education is provided. Due to university facilities’ closures, most courses were conducted online [4]. Furthermore, many facilities restricted the availability of clinical rotation sites during the pandemic. Considering the unprecedented need for future healthcare professionals in the context of a global emergency, maintaining education in this field was essential [5].

The rise of telehealth methods in care services also necessitated training students in novel ways of care delivery [6]. Students expressed concerns about the shortage of clinical rotation sites [7]. Ideally, solutions to be implemented should also benefit patients who are particularly susceptible to disruptions associated with limited access to reliable healthcare during the pandemic [7].

The COVID-19 pandemic also caused serious challenges for interprofessional education (IPE), particularly important in patient-centered healthcare systems where interprofessional collaboration is a necessary prerequisite [8,9]. A Cochrane Database systematic review determined that interprofessional collaboration of physicians and pharmacists is essential for older adults (≥65 years) with increased healthcare needs [10]. Therefore, when developing high-quality virtual programs to be implemented during the pandemic, innovative approaches are indispensable to address the need for IPE of medical and pharmacy students [8,11,12].

## 2. Objective

Because the pandemic overburdened healthcare establishments (including healthcare education facilities), the authors developed and implemented a pilot, remote, experiential IPE project in order to provide medical and pharmacy students with practical learning opportunities while providing care to vulnerable older adults (≥60 years) with limited healthcare access. The project’s goals were to improve student satisfaction with virtual learning and measure patient acceptance of an interprofessional student telehealth education project.

## 3. Patient Involvement and Methods

Authors developed widespread promotional materials (posters, local news services announcements, radio advertisements, and television interviews) for this initiative. The program used the Internet-Telephone Consultation Services (ITCS) available at Poznań University of Medical Sciences (PUMS). The ITCS was introduced by the Department and Chair of Palliative Medicine, University of Medical Sciences, in response to students’ educational needs and the growing demand of older adults for telehealth during the pandemic. The main objective of the ITCS was interprofessional medication management performed by medical and pharmacy students working collaboratively under the supervision of their academic mentors. This service was launched in March 2020 as a part of the Poznań University of Medical Sciences COVID-19 student volunteer initiative (voluntary movement of the students). Consultations were conducted six days a week during working hours (8 a.m.–4 p.m.).

### 3.1. Students Involvement

Only students with previous interprofessional experience and in the last year of their curriculums were recruited for the project. This previous interprofessional experience was gained through participation in any interprofessional electives organized at PUMS in the previous academic years. The medical curriculum (MD) in Poland spans six years, while the pharmacy curriculum (MPharm) is completed in five and a half years. Ordinary curriculums (MD, MPharm) do not include IPE activities within.

In total, 13 students (six medical and seven pharmacy students) were involved in the project. Students were divided into four groups with two or three students per group. Each group contained both medical and pharmacy students. Each group designated a group leader who was responsible for contacting patients prior to the consultation. Two students were not involved in direct telehealth visits and instead helped mentors organize consultations and were available to address patient inquires.

### 3.2. Consultation Process

Patients contacted ITCS using the phone number included in widespread promotional materials. After registration, the student group leader received patient contact information and scheduled an initial phone call. For this initial phone call, consideration was given to the time and means of communication (telephone, WhatsApp^®^, or Messenger^®^) preferred by the patient. The first remote meeting was held to obtain medical history, a comprehensive medication list (along with dosage, time of day of medication administration, dosage form, and frequency, i.e., BID, TID, or QID), the patient’s concerns, and inquiries about pharmacotherapy or chronic conditions management. This meeting was always followed by a mentor-guided discussion during which students presented their individualized patient recommendations and received feedback. A physician and a pharmacist who are members of the European Academy for Medicine of Ageing (EAMA) provided students with substantive support throughout the consultations and handled organizational affairs. These supervisors directly observed students during a series of initial consultations. Once mentors agreed that students were ready to conduct a consultation without direct observation, the teams were allowed to contact patients on their own. Regardless of students’ independence, the mentor-guided discussion always preceded the issuance of the recommendations. The direct mentoring period lasted between two to six observed consultations, depending on the student team’s proficiency and recommendations appropriateness as assessed by the supervising practitioners. In each case, a second meeting was arranged during which students provided the patient with mentor-discussed recommendations along with a brief explanation of proper pharmacotherapy and chronic condition management. Each recommendation was carefully prepared based on the most recent sources and guidelines. Recommendations did not alter prescribed medications’ management directly unless discussed with the patient’s primary care physician if necessary. If discrepancies or inconsistencies of prescribed medications were detected, patient’s primary care physician was notified (as primary care physicians in Poland do not perform comprehensive medication list reviews routinely).

### 3.3. Data Collection

Feedback was collected by a phone call to patients after 4–8 weeks of consultation. This period was established in order to allow patients to consider their overall satisfaction with the recommendations while still being able to recall the experience. Patients rated the experience on a five-point Likert scale in which responses could range from strongly disagree (1) to strongly agree (5). This data was collected by a student who was not involved in the care of the patient to avoid influencing patient feedback. The survey questions were based on patient satisfaction domains from a student-run free clinic study by Lawrence, et al. [13]. These survey questions examined patient interest in future participation in a similar project, patient assessment of the quality of care and concern, and patient perception of the importance of the information provided by the students. In addition, four students (two medical students—male and female; two pharmacy students—male and female) and four patients (two males, and two females) participated in in-depth interviews to better understand the project’s significance. The interviewer was a psychologist who was experienced in performing in-depth interviews. Based on the available literature, the themes to be explored during the in-depth interviews were discussed and established by the members of EAMA (physician, and pharmacist) and the interviewer [14,15,16]. The themes were generated before the interviews and were followed by the psychologist during the sessions. For the students, they included self-assessment, overall satisfaction with the IPE experience, interprofessional communication, roles, and responsibilities within the student groups, interaction with the mentors, skills and knowledge gained, and drug therapy problems encountered during the consultations (Table 1). The interview themes for the patients are presented in Table 2. The interviews were conducted virtually (Microsoft Teams^®^, PUMS intranet—students; telephone—patients). Prior to the interview, the psychologist informed the participant about the purpose of the research, acquired consent for recording, and ascertained the participant was alone. The interviews were audiotaped, transcribed, and anonymized. After each interview, member checking (respondent validation) was performed in order to improve the trustworthiness of the data.

### 3.4. Data Analysis

The questionnaire was evaluated for reliability. Internal consistency was validated with the Cronbach’s alpha coefficient based on the George and Mallery rating score (≥0.9: excellent, <0.9 to ≥0.8: good, <0.8 to ≥0.7: acceptable, <0.7 to ≥0.6: questionable, <0.6 to ≥0.5: poor, and ≤0.5: unacceptable [17]). Analysis of students’ perspectives on the interprofessional education activity as well as detected pharmacotherapy discrepancies was performed by the supervising physician and pharmacist. Extraction of audio transcribed patients’ quotations was performed by two students (who did not perform consultations) working independently. A patient quotation was included in Table 2 when the two students had independently extracted the same sentence from the interview record.

Standards for Reporting Qualitative Research (SPQR) were considered in the preparation of this study [18]. The study was authorized by the Poznań University of Medical Sciences Ethics Committee (Reference Number: KB-544/20).

## 4. Results

To date, 49 consultations have been conducted. In-depth interviews of student participants revealed qualitative outcomes of this initiative. Both medical and pharmacy students stated that the telehealth process allowed them to review material from their didactic coursework. Developing and presenting recommendations strengthened their self-confidence and understanding of patient care skills. Moreover, their ability to conduct a literature review was improved because each consultation demanded appropriate preparation. Real-time consultation enhanced the development of communication and telehealth skills. Furthermore, the interprofessional collaboration of medical and pharmacy students flowed naturally and this project allowed their IPE to continue during the pandemic lockdown. Participating students stated that interprofessional practice gave them a different perspective on the care of the patient as well as developed a holistic approach to the patient. Moreover, they admitted that their trust in the other profession and appreciation for their colleagues were enhanced. Students reported satisfaction with the knowledge, skills, and attitudes acquired from the project. The mentors’ involvement was considered helpful and instructional. Representative problems encountered and detected pharmacotherapy discrepancies or inconsistencies with student perspectives on consultation requirements and acquisitions of collaborative work are presented in Table 1.

**Table 1:** Detected pharmacotherapy management inappropriateness in patients along with categorization of medical and pharmacy students’ perspectives on revised and acquired knowledge.

Category of Detected Drug Therapy Problem (According to Strand et al. [19])	Example	Medical Student’s Perspective	Pharmacy Student’s Perspective
Untreated condition	Arterial hypertension (HTN) (inappropriate antihypertensive treatment scheme)	Revision: HTN treatment, medication indications, combination therapy Acquisition: applied pharmacokinetics of combined medications, possible interactions	Revision: antihypertensive medication list Acquisition: diagnostic approach toward HTN, symptoms and complications recognition
No medical indication	Excessive use of proton pump inhibitor (PPI) (>8 weeks duration without indications)	Revision: symptoms and indications for PPI use Acquisition: long- and short-term consequences of excessive PPI use (interactions)	Revision: mechanism of action of PPI, food–drug and drug–drug interactions Acquisition: indications for PPI use
Inappropriate medication	Nonsteroid anti-inflammatory drug (NSAID) analgesic use and concomitant gastrointestinal condition (gastritis)	Revision: contraindications and precautions to NSAIDs Acquisition: analgesic alternatives, NSAID-related interactions	Revision: NSAIDs and their adverse effects (i.e., gastrointestinal), analgesic alternatives Acquisition: NSAID indications
Too low medication dosage	Poorly controlled asthma	Revision: guideline-based (GINA, GOLD) treatment and recognition of poorly controlled asthma Acquisition: steroids and add-on medications	Revision: examples of anti-asthmatic medications and their indication Acquisition: asthma symptoms, staging, and exacerbating factors, recognizing reliable guidelines
Too high medication dosage	Too high metformin dose (prediabetic state)	Revision: diabetes and prediabetes diagnostic criteria, treatment schemes, adverse effects, and monitoring parameters Acquisition: metformin dosage	Revision: metformin indications and dosage Acquisition: prediabetes and diabetes diagnostic criteria and symptoms
Adverse effect	Allergic reaction to herbal dietary supplement)	Revision: differentiating possible exanthema causes (dermatological symptomatology) Acquisition: adverse effects of dietary supplements	Revision: indications for the dietary supplements Acquisition: dermatological symptomatology
Non-adherence	Statin withdrawal	Revision: positive cardiovascular (CV) benefits of statins based on European Society of Cardiology guidelines Acquisition: pharmacokinetics of statins	Revision: statins’ pleiotropic effects, mechanism of action Acquisition: importance of medication compliance and ideal cholesterol target based on CV risk category
Interactions	Beta blocker and nondihydropyridine calcium channel blocker (CCB) combination	Revision: hazardous combinations of medications in antihypertensive treatment schemes Acquisition: awareness of interactions	Revision: mechanism of action of antihypertensive medications Acquisition: recognizing symptoms such as bradycardia

**Figure 1:**
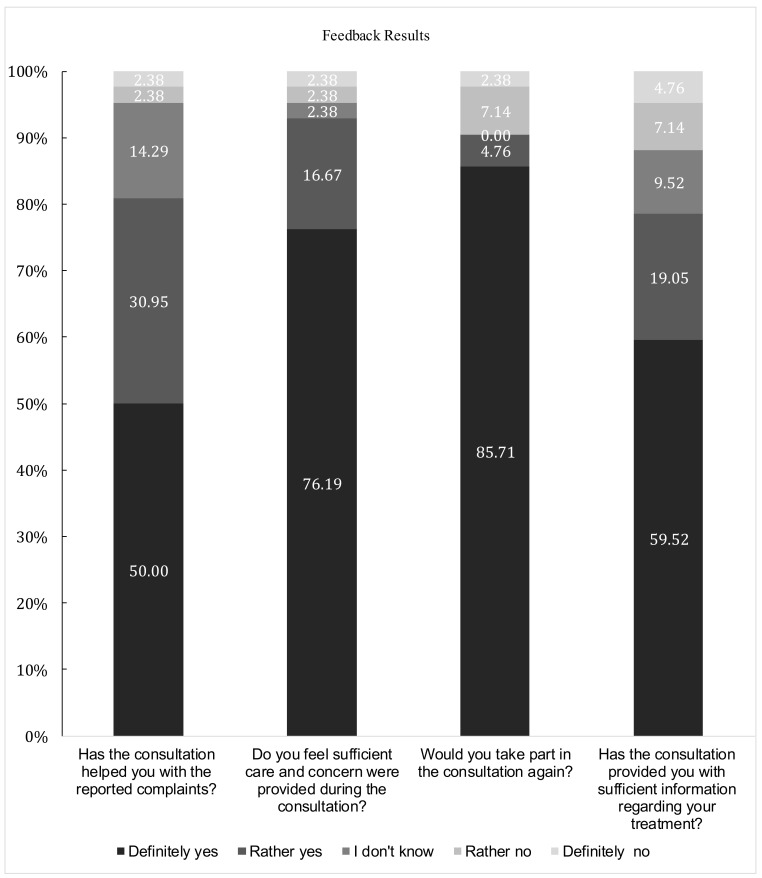
Patient Feedback Results Collected via Telephonic Check-up Approximately 4–8 Weeks after each Consultation. Number and Type of Respondents: 42 Consulted Patients (of 49 in Total; with 7 Patients Remaining Unavailable). Place of Study: Poznań (remotely). Year of Study: 2020.

**Table 2:** Quotations from in-depth interviews of older patients who participated in the project.

Theme	Quotations
Acknowledging the initiative	*“I have read about it in the newspaper. I have thought it must be worth calling.”* *“Local senior club has notified each of our members. I am always keen on benefiting from somebody’s knowledge.”* *“I find it hard to point out one specific source. In my environment it constitutes common knowledge that this initiative was designed for assistance of the older patients.”*
Expectations and motivation toward the initiative	*“At the time I needed help. I was curious if any other methods of my condition management are available.”* *“I often participate in the events organized by our local University of the Third Age. It has always been pleasing.”* *“I have lots of medications administered. I wanted to consult it back then.”* *“I stay conscious of my health. I am interested in both natural and academic medicine. I stay alert and verify my treatment.”*
Attitude toward online consultations performed by medical and pharmacy students	*“I find these consultations very helpful. I really appreciate the fact I had an opportunity to revise my medication list. Students remained investigative and caring—they called me several times to make sure I understood their recommendations correctly.”* *“I think it is better to clarify your concerns with professionals rather than search for answers on your own. Especially when it involves health-related topics. Thanks to them I returned to prescribed medications I had dechallenged on my own. Earlier, I didn’t acknowledge importance of my therapy.”* *“It remains crucial that recommendations were discussed with their tutors. However they are about to graduate they still may lack practical knowledge. I also think that remote formula may be problematic for some people let alone restrictive in terms of patient examination. Thankfully, students offered different means of communication depending on preferences—computer or telephone.”*
Attitude toward interdisciplinary characteristics of performed consultations	*“It is certainly going to help them in their working life. It is important to learn how to work with different professions.”* *“They were supplementing each other. I have talked to both medical and pharmacy students equally.”* *“I have personally felt well-cared.”* *“Consulting real-life patients must have been challenging for both students.”*
Post-pandemic perspective of the initiative and suggestions for expansion	*“It could serve its purpose. Doctors often do not have enough time to spare for such detailed explanations and discussions.”* *“I know that some inquiries do not necessitate an appointment in the clinic. Many inquiries are often trivial—students could definitely cope with it. I would personally appreciate settlement of working hours.”* *“This service should be advertised and expanded. Both students and older patients can benefit from it.”*

Feedback results were collected from 42 patients, with seven patients remaining unreachable. The Cronbach alpha value for the questionnaire was 0.81, suggesting good internal consistency. Patient feedback results are presented in Figure 1.

Overall older adults were open to participating in the program and appreciated the opportunity to do so. Quotations from in-depth interviews conducted among four older patients who participated in the project were collected in Table 2.

## 5. Discussion

The qualitative outcomes of in-depth interviews among student participants revealed that this remote education project proved satisfactory for both medical and pharmacy students in terms of interprofessional and experiential learning. The findings from the interviews are aligned with the literature. Cooke, et al. showed that medical and pharmacy students who participated in simulated IPE activities gained an appreciation for each profession’s impact on the patient’s therapy [20]. According to Khalili et al. online IPE helped students understand the roles of every member of the healthcare team and experience hard-to-teach concepts like teamwork, leadership, and readiness to collaborate during emergencies [8]. Villela et al. reported that students have contributed to the control of the pandemic using acquired health knowledge and virtual platforms [21]. Moreover, Bautista et al. revealed a beneficial impact of an interprofessional rotation on the collaboration of medical and pharmacy students through telehealth outreach to vulnerable patients during the COVID-19 pandemic [11].

Medical and pharmacy students are distinguished by their specialized knowledge. Medical students’ clinical orientation and the pharmacologic knowledge of pharmacy students act in synergy to build a foundation for interprofessional, comprehensive care [22]. This is particularly visible when medical and pharmacy students’ perspectives are compared (Table 1).

Patient satisfaction within this IPE project is notable, with 92.68% of surveyed patients giving positive responses when asked if sufficient care and concern were provided during the interprofessional consultations. Moreover, 80.59% of surveyed patients stated that the consultations helped them with their reported issues. Nearly 80% of patients felt that the provided health information was sufficient.

While developed to maintain patient care and student education during the pandemic, this program will likely continue post-pandemic, based on its positive outcomes. The reality of pandemic education demands innovative and substantive educational offerings to replace live clinical rotations. Implementing this virtual experience at the end of the curriculum allows students to make use of their previous knowledge in a practical setting. This early introduction of interprofessional practice is auspicious for continued professional collaboration. The practical application of pharmacological knowledge in response to problems encountered during geriatric-focused consultations is visible. The study by Kelly et al. suggested that both medical and pharmacy students can gain significantly from collaborative experiences, especially if the implementation of such practice is made in the early stages of their education [23]. Moreover, recommendation issuance might help participants (medical and pharmacy students) overcome their future authority and extreme responsibility when they became independent practitioners.

Older adults were able to operationalize the telehealth interaction, with 90.47% stating they would utilize this service again. The widespread acceptance suggests that this type of care may allow student teams with mentors to improve the healthcare of older adults by improving accessibility, especially in underserved communities. To this extent, Poznań University of Medical Sciences has reached an agreement with a non-profit foundation to expand this voluntary consultation service and include it as part of a nationwide project encompassing remote assistance for the older population. Within this non-governmental organization, students will be able to provide consultations at any time. Moreover, to ensure that more students have the opportunity to gain experience with counseling, the authors developed an elective course incorporating the methods in question.

This easy-to-implement system is adaptable in many countries for interprofessional student education in older care. One international partner, the University of Kentucky College of Pharmacy, has observed this practice and is currently in the very early stages of the development and implementation of a similar project. Projects such as this may also be viewed as a way to provide international exchange between institutions when travel is restricted, or students are unable to gain international experience otherwise.

During this first implementation, the authors uncovered several limitations that will be addressed in the future. Older adults in Poland and elsewhere are less likely to use electronic devices than younger individuals and might be considered harder-to-reach with the use of information technologies. The reasons for this phenomenon might be decreased internet accessibility, hearing loss, and low familiarity with or acceptance of technology [24]. The authors’ experience with the implementation of the pilot study provided valuable insights that will be used to increase the reach to the older adult population (60 years and older). Given the small scale of the pilot project, only a limited number of students had the opportunity to participate, resulting in a relatively small number of patient consultations. With the implementation of the new elective course and the partnership with a non-profit foundation, the authors are optimistic about the expansion of the program to benefit students from Poland and other countries, as well as a larger number of groups of older people.

## 6. Conclusions and Practical Value

Remote consultations of older adults performed by medical and pharmacy students working collaboratively were beneficial for continuity of IPE and management of care of older patients during the COVID-19 pandemic. Continued discussions on further development and wide implementation of such approaches as a long-term solution should be considered.

## Data Availability

The data presented in this study are available on request from the corresponding author. The data are not publicly available due to personal characteristics of the acquired data.
